# 胰岛素抵抗与肺癌临床相关机制的研究进展

**DOI:** 10.3779/j.issn.1009-3419.2024.106.27

**Published:** 2024-10-20

**Authors:** Xiaoyu CHEN, Peng CHEN

**Affiliations:** 300060 天津，天津医科大学肿瘤医院肺部肿瘤内科，国家恶性肿瘤临床医学研究中心，天津市恶性肿瘤临床医学研究中心，天津市肿瘤防治重点实验室; Department of Thoracic Oncology, Tianjin Medical University Cancer Institute & Hospital, National Clinical Research Center for Cancer, Tianjin’s Clinical Research Center for Cancer, Key Laboratory of Cancer Prevention and Therapy, Tianjin 300060, China

**Keywords:** 肺肿瘤, 胰岛素抵抗, 胰岛素样生长因子1受体, 耐药, Lung neoplasms, Insulin resistance, Insulin-like growth factor 1 receptor, Drug resistance

## Abstract

目前全球肺癌的发病率和死亡率均位居恶性肿瘤首位。肺癌的早期诊断、治疗和耐药仍是肺癌管理的难题。研究人员致力于寻找可靠的生物标志物作为肺癌的预测指标或治疗的有效靶点。胰岛素抵抗（insulin resistance, IR）是一种以胰岛素生物活性降低为特征的疾病，会导致胰岛素分泌增加。近年来，越来越多的研究发现IR与癌症的发生和进展之间存在联系，胰岛素/胰岛素生长因子信号通路在其中可能起到关键作用。本文重点阐述IR与肺癌之间的关系，探讨IR对肺癌发生、进展和耐药的影响及作用机制，以期指导新的预测工具和治疗策略的开发，为致力于降低肺癌发病率和死亡率的研究提供新思路。

## Abstract (en)

At present, the incidence and mortality rates of lung cancer rank top among malignant tumors. The early diagnosis, treatment and drug resistance of lung cancer still remain as problems in the management of lung cancer. Researchers are dedicated to identifying reliable biomarkers as predictive indicators or effective therapeutic targets for lung cancer. Insulin resistance (IR), a disorder characterized by reduced biological activity of insulin, leads to increased insulin secretion. In recent years, more and more studies have revealed the association between IR and the occurrence and development of cancer, with the insulin/insulin-like growth factor signaling pathway possibly playing a crucial role. In this article, we will focus on the relationship between IR and lung cancer, explore the impact and mechanism of IR on the development, progression and drug resistance of lung cancer. It may guide the development of new predictive tools and therapeutic strategies, and provide new ideas for research dedicated to reducing the incidence and mortality of lung cancer.

癌症作为21世纪一个重大的公共卫生问题，已成为全人类死亡的主要原因之一。2022年癌症数据^[1]^显示，全球癌症新发病例数约2000万，死亡病例数约970万，其中肺癌新增病例数近250万（占12.4%），死亡人数约180万（占18.7%），肺癌已经成为全球发病率和死亡率最高的恶性肿瘤。肺癌的早期症状隐匿，大多数患者确诊时已是晚期，错失了手术机会，导致预后受损。虽然肺癌的治疗不断取得进展，但获得性耐药不可避免，5年生存率仍不尽如人意。癌症的发生和进展机制十分复杂，涉及多方面因素。越来越多的研究^[2]^发现胰岛素抵抗（insulin resistance, IR）与癌症之间存在关联。

IR是指主要靶组织（如脂肪组织、肝脏和骨骼肌）对生理性胰岛素水平的反应性和敏感性减弱，导致胰岛素作用不足，引起长期的全身性高胰岛素血症^[3]^。胰岛素作用不足会导致信号转导受损^[4]^，在IR状态下胰岛素/胰岛素样生长因子（insulin-like growth factor, IGF）信号通路存在异常，胰岛素分泌增加伴随IGF的分泌增加。IGF与IGF受体结合，诱导受体自身磷酸化，进而磷酸化受体底物，激活下游磷脂酰肌醇-3-激酶（phosphatidylinositol-3-kinase, PI3K）/蛋白激酶B（protein kinase B, Akt）和Ras/丝裂原活化蛋白激酶（mitogen-activated protein kinase, MAPK）信号通路，参与癌症发生和进展的多个过程^[5]^。因此，准确高效地评估IR具有重要意义。正常血糖-高胰岛素钳夹技术是评估IR的金标准^[6]^，但仅适用于实验室测量，不具有普及性。后来，一些简单易获取的指标被开发出来，如胰岛素抵抗稳态模型评估（homeostasis model assessment of insulin resistance, HOMA-IR）^[7]^、甘油三酯-葡萄糖（triglyceride glucose, TyG）^[8]^、TyG-体重指数（TyG-body mass index, TyG-BMI）^[9]^、糖脂代谢指数（glucose-lipid metabolism index, GLMI）^[10]^、IR代谢评分（metabolic score for insulin resistance, METS-IR）^[11]^等，计算公式见表1。探索新的预测生物标志物以及深入了解胰岛素/IGF信号通路（IGF轴）在肺癌细胞形成、增殖、侵袭和耐药中的作用机制对肺癌的诊断和治疗至关重要。本文旨在探讨肺癌IR的机制，提供研究进展综述，加深对IR与肺癌关系的认识，以指导新的预测工具和治疗策略的开发。

**表1:** IR评估指标计算公式

Assessment indicator	Calculation formula
HOMA-IR	FPI (μIU/mL)×FPG (mmol/L)/22.5 or FPI (μIU/mL)×FPG (mg/dL)/405
TyG	ln [TG (mg/dL)×FPG (mg/dL)/2]
TyG-BMI	TyG×BMI (kg/m^2^)
GLMI	TyG×BMI (kg/m^2^)×TC (mg/dL) /HDL-C (mg/dL)
METS-IR	ln [2×FPG (mg/dL)+TG (mg/dL)]×BMI (kg/m^2^)/ln [HDL-C (mg/dL)]

IR: insulin resistance; HOMA-IR: homeostasis model assessment of insulin resistance; TyG: triglyceride and glucose index; GLMI: glucose-lipid metabolism index; METS-IR: metabolic score for insulin resistance; FPI: fasting plasma insulin; FPG: fasting plasma glucose; TG: triglyceride; BMI: body mass index; TC: total cholesterol; HDL-C: high-density lipoprotein cholesterol.

## 1 IR与癌症之间的关联

IR引起糖脂代谢紊乱，易导致肥胖、糖尿病、代谢综合征、高胰岛素血症等疾病的发生^[12,13]^。患有IR及相关代谢疾病的患者发生癌症的风险更高。荟萃分析^[8]^表明，较高的TyG指数可能会增加癌症风险，HOMA-IR值较高的患者患乳腺癌^[14]^、子宫内膜癌^[15]^、前列腺癌^[16]^、结直肠癌^[17]^的风险也更高。有研究^[18]^发现，参与IR的小分子RNA（miR-29b）能够通过靶向Ras/MAPK和PI3K/Akt信号通路促进子宫内膜癌的肿瘤血管生成。胰岛素水平升高在肿瘤前和肿瘤细胞中起生长刺激作用^[19]^，荟萃分析^[16,20]^也证实血清胰岛素和IGF-1水平升高与乳腺癌、前列腺癌风险之间存在很强的相关性；高胰岛素血症可能通过影响肝脏性激素结合球蛋白的产生和分泌，增加雌二醇和睾酮的生物利用度，影响生殖相关肿瘤的发生和进展^[21]^。此外，IR还可能与胃癌发生和进展^[22]^以及甲状腺癌发生和复发风险增加显著相关^[23,24]^。一项荟萃分析^[25]^表明代谢综合征患者肝细胞癌的发生率较高。

最近的两项荟萃分析均支持癌症患者存在明显的IR^[2,26]^，一些已知的肿瘤分泌因子，如肿瘤坏死因子α、白细胞介素6（interleukin 6, IL-6）等，也与IR有关^[27,28]^。IR可能是癌症相关代谢功能障碍的主要因素，而这又增加了癌症复发和死亡的风险^[26]^。IR与多种癌症的发生和进展之间存在相关性，越来越多的研究也致力于探索IR与肺癌之间的临床关联。

## 2 IR与肺癌的流行病学研究

起初Petridou等^[29]^在雅典人群中进行了一项病例对照研究，通过测定HOMA-IR来评估IR与肺癌之间的联系，结果发现HOMA-IR是肺癌的独立危险因素。Argirion等^[30]^对芬兰一个大型男性吸烟队列进行了前瞻性研究，在调整混杂因素后，胰岛素和HOMA-IR与肺癌风险呈正相关；剔除糖尿病患者后相关性保持不变。Yan等^[31]^首次在中国人群中评估了非小细胞肺癌（non-small cell lung cancer, NSCLC）风险与TyG指数的关系，发现NSCLC的发病率随着TyG指数的升高而升高，TyG指数在不同组织病理学类型或肿瘤原发灶-淋巴结-转移（tumor-node-metastasis, TNM）分期中并无差异。然而，王丽杰等^[32]^提出了相反的发现，他们在无癌症病史的白人队列中没有观察到TyG指数与肺癌风险之间的关联。王国庆等^[11]^利用英国生物样本库中无癌症史的人群（白种人381,951例，非白种人13,353例）数据，探究METS-IR与肺癌风险之间的关联；之后又从基因层面分析METS-IR与肺癌之间有无因果关系。结果发现METS-IR与肺癌风险显著相关，而基因预测的METS-IR与肺癌之间不存在因果关系。这表明，METS-IR可以作为肺癌的生物标志物，但不直接导致肺癌的发生。Liu等^[3]^对此前的文献进行荟萃分析，结果证实IR与肺癌之间有很强的相关性。此外，一项回顾性研究^[6]^发现较高的TyG-BMI指数与晚期NSCLC患者的总生存期和无进展生存期恶化有关，这种关联在吸烟者和C反应蛋白水平较高的患者中更明显。

综上所述，虽然IR与肺癌之间的关系存在一些争议，但是绝大多数研究支持IR与肺癌发展之间的正向关联。IR预测指标可能是肺癌预测的潜在生物标志物。

## 3 IR与肺癌机制的探索

IR是一种以胰岛素生物活性降低为特征的疾病，导致血糖升高和胰岛素分泌增加。胰岛素的分泌增加伴随IGF的分泌增加，且IGF-1受体（IGF-1 receptor, IGF-1R）的表达依赖于肿瘤微环境（tumor microenvironment, TME）中的葡萄糖浓度^[33]^。在高糖环境中肿瘤细胞对胰岛素和IGF刺激的敏感性增加，激活胰岛素受体（insulin receptor, InsR）和IGF-1R，最终可能促进肺癌细胞的增殖、侵袭、转移、上皮间充质转化（epithelial to mesenchymal transition, EMT）和耐药^[5]^。其中，IGF轴是联系IR和癌症的关键。

### 3.1 IGF轴

IGF轴由三类分子组成：（1）配体：胰岛素、IGF-1、IGF-2；（2）受体：InsR、IGF-1R、IGF-2受体（IGF-2 receptor, IGF-2R）；（3）载体：胰岛素样生长因子结合蛋白（insulin-like growth factor-binding proteins, IGFBPs）^[34]^。InsR和IGF-1R属于受体酪氨酸激酶家族，而IGF-2R缺乏激酶活性，主要通过受体介导的内吞作用和溶酶体降解去除IGF-2^[35]^。InsR存在A和B两种亚型，InsR-A与胰岛素和IGF-2结合，介导有丝分裂；InsR-B在生理浓度下与胰岛素结合，参与葡萄糖代谢。与配体结合后受体的酪氨酸激酶结构域被激活，促使受体底物磷酸化，触发多个下游信号通路，包括SH2结构域蛋白（Src homology 2 domain-containing protein, SHC）启动的Ras/MAPK通路和胰岛素受体底物（insulin receptor substrate, IRS）启动的PI3K/Akt通路，参与癌症发生和进展过程^[36]^。IGF轴上的三类分子均被发现与肺癌之间存在一定关联，下面将展开进行介绍。

#### 3.1.1 IGF-1/2和IGF-1R

IGF-1R主要与IGF-1和IGF-2结合，IGF-1/2和IGF-1R过表达参与肿瘤相关巨噬细胞（tumor-associated macrophages, TAMs）极化、EMT和免疫抑制等过程，促进肺癌细胞的形成、增殖、转移和抗凋亡。

已有研究^[37]^报道IGF-1在肺组织细胞的发育和分化中具有重要意义，IGF-1/IGF-1R信号异常可能介导肿瘤形成，增加肺癌的发生风险。IGF-1R信号通路可以诱导78-kDa葡萄糖调节蛋白（78-kDa glucose regulated protein, GRP78）转位到质膜，促进TAMs向M2极化，同时阻断IGF-1和敲除TAMs中的GRP78可抑制M2巨噬细胞诱导的促癌作用^[38]^。IGF-1R在诱导EMT中也发挥作用，先前提出了IL-6/IGF-1R自分泌环的存在，IL-6可以刺激IL-6本身以及IGF-1/2的产生，进而激活STAT3和IGF-1R，诱导肺癌（PC9）细胞的EMT^[39]^。有研究^[40]^发现二甲双胍能够通过调节IGF-1表达诱导NSCLC（H3122）细胞的细胞周期停滞和凋亡。此外，IGF-2通过降低树突状细胞的抗原处理能力、参与Treg细胞的增殖、刺激巨噬细胞向M2极化和维持癌细胞干性，发挥免疫抑制作用^[41]^。外周高胰岛素血症以及自分泌/旁分泌IGF-2也可以通过上调程序性死亡配体-1（programmed death-ligand 1, PD-L1）的表达来支持肺癌细胞免疫逃逸^[41]^。

#### 3.1.2 IGFBPs

IGFBPs是一类具有复杂功能的循环蛋白，作为载体蛋白参与IGF转运，调节IGF的活性，IGFBP1-IGFBP6对IGF具有较高亲和力^[35]^。IGFBP1在NSCLC患者血液中高表达，敲除IGFBP1抑制肺腺癌细胞的生长^[42]^。此外，IGFBP1与肺癌患者的转移和复发相关。在空间受限迁移期间，肿瘤细胞中IGFBP1的表达上调，IGFBP1通过抑制AKT1介导的线粒体超氧化物歧化酶2（superoxide dismutase 2, SOD2）磷酸化来增强SOD2活性，降低受限细胞中线粒体活性氧的水平，抑制肺组织血管中的细胞凋亡，促进癌细胞转移^[43]^。IGFBP1高表达提示肺癌患者预后不良，这可能与中性粒细胞铁死亡引起的肿瘤免疫抑制有关^[42]^。IGFBP1或许可用作NSCLC的预后指标，但靶向IGFBP1能否有效治疗NSCLC还有待确定。IGFBP2在各种肿瘤中过表达^[35]^，可能参与细胞耐药过程。IGFBP2的过表达促进黏着斑激酶磷酸化，导致NSCLC细胞对达沙替尼产生耐药性^[44]^。低氧环境下骨髓间充质干细胞来源的IGFBP2也可以激活IGF-1R促进肺腺癌细胞对厄洛替尼耐药^[45]^。

IGFBP3是含量最高的IGFBP，对IGF-1的亲和力高于IGF-1R^[5]^，是IGF-1的主要载体。目前普遍的观点认为IGFBP3通过高亲和力结合IGF-1，减弱IGF/IGF-1R相互作用，抑制IGF信号传导，从而产生抗肿瘤作用。多项研究^[46,47]^表明，IGFBP3过表达在体内外促进肺癌细胞凋亡、抑制细胞生长并增强NSCLC细胞对顺铂的敏感性。IGFBP3还可以独立于IGF/IGF-1R通路发挥作用，IGFBP3与透明质酸（hyaluronan, HA）结合，阻断HA-CD44的信号传递，以p53依赖性方式诱导细胞凋亡^[48]^。IGFBP3介导波形蛋白与泛素连接酶FBXL14复合物的形成，导致波形蛋白的蛋白酶体降解，抑制EMT过程和癌细胞转移^[49]^。然而，也有研究提出相反的结论，有报道^[47]^称IGFBP3过表达与肺癌细胞侵袭和转移增加以及肿瘤患者预后不良有关，还可能介导肺腺癌的脑转移^[50]^。目前对于IGFBP3的认识仍然不够充分，虽然多数研究支持IGFBP3的抗癌作用，但不排除IGFBP3在调节肺癌发展进程中具有双重作用的可能，也可能存在尚未发现的通路混淆了IGFBP3的作用，需要更深入的研究来解释这一争议。

肺癌患者血清中IGFBP4的水平也会升高，IGFBP4的上调可能与NSCLC细胞凋亡和生长抑制相关^[51]^。IGFBP5也可以竞争性结合IGF-1，阻断IGF-1R的信号传导，IGFBP5的表达受谱系特异性转录因子Achaete-scute同源物1（Achaete-scute homolog 1, ASCL1）的调节，敲低ASCL1后IGFBP5表达下调，导致IGF-1R信号通路过度激活，产生促癌作用^[52]^。有研究^[53]^发现在NSCLC样本中IGFBP6的表达下调，IGFBP6可能通过激活程序性细胞死亡来抑制NSCLC细胞生长。此外，还发现了IGFBP7可能在表皮生长因子受体酪氨酸激酶抑制剂（epidermal growth factor receptor tyrosine kinase inhibitors, EGFR-TKIs）耐药的肺癌细胞系中表达上调^[35]^。不同于IGFBP1-6，IGFBP7对IGF亲和力较低，但对胰岛素特异性亲和，在IGF信号通路中可能发挥一定的调节作用，但IGFBP7在实体瘤中的作用并不一致^[54]^，机制尚不清楚。

综上，IGFBPs可以通过IGF依赖性或非依赖性方式发挥作用，介导肺癌细胞的增殖、转移和获得性耐药。IGFBPs可能具有抑癌和促癌的双重作用，但针对不同IGFBP的研究文献数量较少，证据力度较弱。此外，多种IGFBPs在参与细胞调节过程中是发挥独立作用还是相互作用也不清楚。因此，需要更系统、全面的研究进行深入探索。

### 3.2 IRS

IRS是介导IGF-1R信号转导功能的细胞质接头蛋白，协调细胞外信号向细胞内传递，从而激活PI3K/Akt和Ras/MAPK信号通路，调节细胞生长、代谢和增殖^[55]^。IRS蛋白家族由IRS1-6六个成员组成，目前已经发现IRS1、IRS2和IRS4与肺癌之间存在关联。IRS1参与糖酵解过程，并在维持肿瘤细胞的存活和增殖中发挥重要作用^[56]^。IRS1的过表达加速NSCLC细胞的增殖和迁移，敲低IRS1可以降低PI3K/Akt信号通路的活性，显著抑制NSCLC细胞的生长，促进细胞凋亡^[57]^。研究^[56]^显示，在NSCLC组织中IRS1和PD-L1的表达呈正相关，且PD-L1表达受到IRS1下游信号通路的调控，因此推测IRS1可能参与PD-L1的表达并介导NSCLC的免疫逃逸过程。IRS2高表达与肺腺癌患者的总生存期降低显著相关^[58]^。在分子水平上IRS2受到多种微小RNA（microRNA, miRNA）的调节，如miR-7^[59]^、miR-338-3p^[60]^、miR-613^[61]^，环状RNA（circFAT1、circ_0000003、circFAM126A）通过海绵化特定的miRNA上调IRS2，激活下游信号通路，最终促进肿瘤进展。有研究^[62]^发现IRS4在NSCLC中过表达，通过激活PI3K/Akt和Ras-MAPK信号通路促进NSCLC细胞的致癌活性，敲除IRS4基因可以显著抑制肺癌细胞的增殖、迁移，还可以抑制体内肿瘤的生长，并削弱EGFR-TKIs耐药的PC9细胞对吉非替尼的耐药性。这些发现为肺癌预后提供了新的生物标志物，也为肺癌治疗和克服耐药性提供了潜在靶点。

## 4 IGF轴参与肺癌耐药性的发生

化疗、靶向治疗和免疫治疗是肺癌药物治疗的三大主流手段，但治疗后获得性耐药的发生不可避免，严重损害了患者的预后。近来，越来越多的研究发现IGF轴上的一些分子在介导肺癌细胞耐药的过程中也发挥一定作用。

### 4.1 化疗

IGF/IGF-1R信号通路的激活促进细胞增殖，抑制细胞凋亡，改变药物靶标，并增加转运蛋白的表达以降低细胞内药物浓度，这些都会导致肿瘤细胞对化疗药物耐药^[5]^。Zheng等^[63]^发现环状RNA HC0074027水平与NSCLC对多西他赛、顺铂和紫杉醇的耐药密切相关，产生耐药的机制是HC0074027通过抑制miR-379-5p的活性促进IGF-1的表达，进而导致NSCLC的耐药性。一项针对NSCLC顺铂耐药的研究^[64]^表明，使用人重组IGFBP3或抑制IGF-1R时耐药性可以被逆转。IGFBP3水平升高会抑制IGF-1R-PI3K/Akt通路，增加NSCLC肿瘤细胞对顺铂的敏感性^[5]^。有研究^[48]^发现，酪蛋白激酶可以磷酸化IGFBP3，阻断其与HA的结合，激活HA-CD44信号传导，导致顺铂耐药，这一过程与IGF-1R无关。

### 4.2 靶向治疗

IGF通路与NSCLC细胞对EGFR-TKIs的耐药性有关^[35]^。Guerard等^[65]^证明吉非替尼诱导IGF-1R和双调蛋白在肺腺癌细胞内再分布，促进双调蛋白/IGF-1R复合物的形成，允许IGF-1R的核转运，促使细胞周期依赖性激酶抑制剂的积累和G_1_期阻滞，抑制细胞凋亡，导致吉非替尼耐药。NSCLC经常对奥希替尼产生耐药性。奥希替尼暴露可能刺激转录因子FOXA1的表达，增加IGF-1R的蛋白表达和磷酸化，激活信号传导通路以产生耐受性^[66]^。最近一项研究^[67]^在已建立的和患者来源的肺癌细胞中均证明IGF-2自分泌介导IGF-1R通路激活是肺癌中奥希替尼获得性耐药的机制。Hayakawa等^[68]^获取的临床患者标本也为IGFBP3表达下调和IGF-1R激活提供了证据，IGFBP3表达缺失激活IGF-1R也可能是奥希替尼耐药的机制之一。有研究^[54]^发现在奥希替尼耐药的NSCLC细胞和肺癌患者中IGFBP7的表达显著增加，IGFBP7过表达能够促进耐药后的肿瘤进展，而IGFBP7低表达可以恢复EGFR突变细胞对奥希替尼的敏感性，这表明IGFBP7可能是EGFR突变型NSCLC奥希替尼耐药后的潜在治疗靶点。

### 4.3 免疫治疗

肺癌细胞中IGF-1或IGF-1R的高表达与晚期患者对抗程序性死亡受体-1（programmed cell death protein 1, PD-1）和PD-L1治疗的耐药性相关。在NSCLC临床前模型的研究^[69]^中，抑制IGF轴可使肿瘤对PD-1阻断敏感，增强PD-1/PD-L1抑制剂的抗肿瘤作用。IGF轴参与T细胞活化、增殖和存活的复杂过程，抑制或下调IGF-1R可与PD-1阻断协同作用，导致TME中Treg细胞水平降低，CD8^+ ^T细胞水平升高，逆转T细胞耗竭并恢复抗肿瘤免疫^[69]^。此外，有研究^[70]^显示，在高血糖状态下吸烟通过增强TAMs的葡萄糖摄取，上调IGF-2的表达，激活肺癌细胞中的InsR，促进其核易位，核InsR与核磷蛋白结合，介导CD274启动子激活，刺激PD-L1的表达，促进肺癌的进展。遗憾的是，虽然上述研究支持IR相关信号通路中的某些分子参与肺癌细胞的耐药过程，但目前没有直接的证据表明IR是否与肺癌耐药有关，仍需要进一步的研究来解答这一问题。

## 5 结语

IR和肺癌进展之间存在千丝万缕的联系。虽然IR的测量指标层出不穷，但临床上还没有公认的IR的阈值^[7]^，仍需要进一步研究解决这一问题。利用简单易获取的血糖（如空腹血糖、胰岛素）和血脂（如总胆固醇、甘油三酯、高密度脂蛋白胆固醇）等指标量化IR在一定程度上可以预测肺癌风险及预后。鉴于IGF轴参与肺癌的复杂机制，IGF通路的各类分子也应该是肺癌生物标志物的潜在候选者，已有学者^[71]^提出IGFBP1可能是吸烟相关肺癌的预测生物标志物，未来应探索更多简单可靠的预测标志物，准确高效地识别肺癌高危人群，降低肺癌的死亡率。

IGF-1R在肺癌细胞耐药机制中发挥重要作用，免疫组化法检测IGF-1R已经被用于NSCLC患者的临床治疗^[72]^。IGF-1R信号通路上的各个分子为开发新的肺癌治疗措施以及克服获得性耐药提供了有前途的靶点。目前已获得的新型小分子激酶抑制剂LL6，可以靶向IGF-1R、Src和AXL，多靶点抑制体内外NSCLC的生长、迁移以及各种耐药细胞系的集落形成能力，且毒性较低^[73]^，这为开发IGF通路抑制剂提供了希望，也为抗癌治疗提供了新的途径。IGF通路抑制剂与细胞毒性药物、EGFR-TKIs或PD-1/PD-L1抗体的联合使用可能是未来治疗晚期肺癌的潜在手段。
